# Evaluating Model Fit of Measurement Models in Confirmatory Factor Analysis

**DOI:** 10.1177/00131644231163813

**Published:** 2023-04-02

**Authors:** David Goretzko, Karik Siemund, Philipp Sterner

**Affiliations:** 1 Ludwig-Maximilians-Universität München, Germany; 2 Utrecht University, The Netherlands

**Keywords:** confirmatory factor analysis, model fit, review, fit indices, dynamic cutoffs

## Abstract

Confirmatory factor analyses (CFA) are often used in psychological research when developing measurement models for psychological constructs. Evaluating CFA model fit can be quite challenging, as tests for exact model fit may focus on negligible deviances, while fit indices cannot be interpreted absolutely without specifying thresholds or cutoffs. In this study, we review how model fit in CFA is evaluated in psychological research using fit indices and compare the reported values with established cutoff rules. For this, we collected data on all CFA models in *Psychological Assessment* from the years 2015 to 2020 $\left(N_{\mathrm{Studies}}=221\right)$ . In addition, we reevaluate model fit with newly developed methods that derive fit index cutoffs that are tailored to the respective measurement model and the data characteristics at hand. The results of our review indicate that the model fit in many studies has to be seen critically, especially with regard to the usually imposed independent clusters constraints. In addition, many studies do not fully report all results that are necessary to re-evaluate model fit. We discuss these findings against new developments in model fit evaluation and methods for specification search.

## Introduction

Developing measurement models for psychological constructs is always challenging. For questionnaire development and test construction, researchers conduct several factor analyses to carve out the latent variables representing a psychological concept (e.g., Fabrigar et al., 1999). Usually, exploratory factor analysis (EFA) is used to explore an item set associated with a construct (or specifically designed to measure a certain psychological variable) and subsequently refine it. After several rounds of EFAs applied to different item sets and samples, researchers come up with a hypothesized factor model that describes how the latent variables are measured by the manifest indicators. These hypothesized factor models that are usually built around an assumption of independent clusters (i.e., that each indicator only measures one latent factor and has no substantial cross-loading on a second factor) can be tested in a confirmatory setting with a confirmatory factor analysis (CFA). CFA evaluates whether an assumed relationship among manifest indicators and latent factors is in line with the empirical data by testing whether the model-implied covariance structure reproduces the empirical covariance matrix (or resembles it very strongly). When conducting a CFA, the researcher specifies the number of latent factors, which manifest indicators are allowed to load on which latent factor (i.e., which loadings are freely estimated and which are constrained to be zero), whether between-factor correlations are allowed and whether there are any correlations among the residuals of the indicators. These specifications are based on empirical findings in previous studies (often based on EFA) as well as theoretical considerations (for a thorough introduction to CFA, see, for example, Brown, 2015). Since previous findings might support different models or contradict theoretical implications, different candidate models have to be compared. Hence, CFA users need to know which models fit their data and which model is the most plausible given their data. While there is a global model $\chi^{2}$ -test that tests whether the model-implied covariance Σ(Θ) is exactly the same as the population covariance matrix Σ , its null hypothesis ( Σ=Σ(Θ) ) hardly ever holds for empirical data (Bentler, 2007). Hence, the dichotomy of this global model test (model-implied covariance matrix equals the population covariance matrix or differs from it) is often replaced with a more nuanced perspective by taking so-called fit indices into account which quantify the level of model (mis-)fit (e.g., Schermelleh-Engel et al., 2003). In other words, instead of a clear decision on whether a proposed model can be seen as the true population model, the model misfit is quantified (e.g., MacCallum, 2003).

In this study, we review the use of CFA in psychological research with a focus on model selection and the assessment of model fit (particularly the use of model fit indices). In doing so, we also reevaluate the model fit of published studies (where possible) using the *Dynamic Fit Index Cutoffs* approach by McNeish and Wolf (2021) as well as the *ezCutoffs* approach (Schmalbach et al., 2019). Based on our findings, we discuss the need for methodological rigor in the model evaluation as well as caveats that hinder the development of measurement models that better fit empirical data.

### Fit Indices

A large set of fit indices have been developed to quantify the goodness of fit or the deviance from the perfect model fit. The latter are error-focused measures that quantify the difference between an empirical covariance matrix S and the estimated model-implied covariance matrix $\Sigma (\hat{\Theta })$ . The two most common measures of this kind are the *Root Mean Square Error of Approximation* (RMSEA) and the *Standardized Root Mean Square Residual* (SRMR).

The RMSEA (Steiger, 1998) quantifies the error of the approximate fit, that is, it replaces the “exact fit”-null-hypothesis of the global $\chi^{2}$ -test with a hypothesis of an approximate or “close” fit. In doing so, the difference in the minus two log-likelihood between the tested model and a saturated model^1^ for the empirical data $\chi^{2}$ is compared to its expected value given that the proposed model is not misspecified df (this expected value equals the degrees of freedom of the tested model).

$$\mathrm{RMSEA}=\sqrt{\max\left(\frac{\chi^{2}-\mathrm{df}}{(N-1)\mathrm{df}},0\right)},$$

with N being the sample size. When the proposed model is not misspecified, the fraction within the square root has the expected value of zero, which is why an RMSEA close to zero indicates a “close” fit (RMSEA cannot be negative as negative deviations from the expected value df are set to zero, so that the square root can always be applied).

The SRMR is also a “badness of fit” measure as it quantifies the averaged squared differences between each bivariate empirical correlation and the respective model-implied counterpart (Hu & Bentler, 1998). Hence, the best possible value is zero indicating a perfect reproduction of the empirical correlation matrix, while higher SRMR values reflect a poorer model fit. By standardizing the residuals using the standard deviations of the respective manifest items the SRMR is scaled (compared with the *Root Mean Square Residual* [RMSR] index by Jöreskog & Sörbom, 1996) and its maximum possible value is one.

$$\mathrm{SRMR}=\sqrt{\frac{\sum_{i=1}^{p}\sum_{j=1}^{i}{\left[\frac{s_{\mathrm{ij}}-{\hat{\sigma }}_{\mathrm{ij}}}{s_{i}s_{j}}\right]}^{2}}{p(p+1)}},$$

with p being the number of manifest variables, $s_{\mathrm{ij}}$ being the empirical covariance between the *i*th and *j*th indicator, ${\hat{\sigma }}_{\mathrm{ij}}$ being the model-implied covariance between the *i*th and *j*th indicator, and $s_{i}$ and $s_{j}$ being the empirical standard deviation of the *i*th and *j*th indicator, respectively.

Besides these measures of model misfit, goodness-of-fit measures such as the *Goodness-of-Fit Index* (GFI; Jöreskog & Sörbom, 1984, as cited in Mulaik et al., 1989), the *Normed Fit Index* (NFI; Bentler & Bonett, 1980), the non-NFI that is also known as the Tucker–Lewis Index (TLI; Tucker & Lewis, 1973), or the *Comparitive Fit Index* (CFI; Bentler, 1990) exist. For these indices, a model comparison between the proposed model and a baseline model is conducted. The GFI quantifies how much better the proposed model fits the data compared to a null model as a baseline model (i.e., a model that can be described as a “no-factor null model”—it can also be interpreted similar to a coefficient of determination as the proportion of the variance/covariance that can be explained by the model, for more information on this and different versions of the GFI, see Mulaik et al., 1989). A value of one indicates that the proposed model provides the biggest improvement possible over the baseline model and is able to fit the data perfectly, while a value of zero means that the proposed model has no explanatory value.

$$\mathrm{GFI}=1-\frac{\chi^{2}}{\chi_{\mathrm{Null}}^{2}}.$$

The NFI follows the same idea. Contrary to the GFI, the baseline model used to calculate the NFI is the so-called independence model which assumes the error variance to be zero and no existing associations among the observed variables (i.e., only the variances of the observed variables are estimated, so the independence model is basically a diagonal matrix with all off-diagonal elements—the covariances—being zero). Accordingly, the NFI can be written as

$$\mathrm{NFI}=1-\frac{\chi^{2}}{\chi_{\mathrm{Independence}}^{2}}.$$

The TLI compares the proposed model to the independence model as well.

$$\mathrm{TLI}=\frac{\frac{\chi_{\mathrm{Independence}}^{2}}{df_{\mathrm{Independence}}}-\frac{\chi^{2}}{\mathrm{df}}}{\frac{\chi_{\mathrm{Independence}}^{2}}{df_{\mathrm{Independence}}}-1}.$$

Its values normally range from zero to one, but as it is not normed, values outside this range that are less intuitive might occur. A TLI greater than one is possible (i.e., a value of one does not mean a perfect fit, contrary the other goodness of fit measures) and can be interpreted as indicative of a very good model fit.

The CFI also relies on the independence model for comparison. Contrary to the NFI or GFI and comparable to the TLI, the degrees of freedom (i.e., the expected value if the model is correctly specified) are taken into account.

$$\mathrm{CFI}=1-\frac{\max[(\chi^{2}-\mathrm{df}),0]}{\max[(\chi_{\mathrm{Independence}}^{2}-df_{\mathrm{Independence}}),(\chi^{2}-\mathrm{df}),0]}.$$

The CFI, just like both GFI and NFI,^2^ becomes one if the proposed model fits the data perfectly and zero in a worst-case scenario where it is not superior to the baseline model.

While these and other fit indices are frequently applied to assess the model fit in structural equation modeling (SEM) in general, and in CFA in particular, several studies found them to be dependent on nuisance parameters and the underlying data conditions. Hence, their ability to detect model misspecifications does not only depend on the type of misspecification (e.g., Hu & Bentler, 1998) but also on the sample size (e.g., Ainur et al., 2017; Fan & Wang, 1998), the size of the loading parameters (e.g., Heene et al., 2011), the number of indicators per factor (e.g., Shi et al., 2019) or the overall model complexity (e.g., Marsh et al., 1996), the amount of missing data (e.g., Fitzgerald et al., 2021), and the estimation method (e.g., Fan et al., 1999; Xia & Yang, 2019). Crucially, poorly measured indicators (Heene et al., 2011; McNeish et al., 2018) as well as untreated missing data (Fitzgerald et al., 2021) may disguise model misfit and substantial misspecifications as fit indices show fallaciously good values indicating acceptable model fit.

Another problem that arises with the usage of fit indices is the challenge to determine which values are indicative of an “acceptable,” a “good” or an “excellent” model fit. When comparing candidate models and selecting a final model, relying on different fit indices is less problematic. However, when the absolute fit of a specific candidate model is evaluated, researchers usually look for cutoff values that categorize the goodness of fit. Often simple cutoff rules that are based on simulation studies with very narrow data conditions (e.g., Hu & Bentler, 1999) are used beyond the scope of the study they are derived from. Marsh et al. (2004) describe the dangers of overgeneralizing the results of these simulation studies and call for a more thoughtful handling of suggested cutoffs.

### Tailored Cutoffs for Model Evaluation

To both accommodate the desire for categorical decisions, for example, labeling a model’s fit as “good/appropriate” or “bad,” and overcome the limitations of narrow data conditions and model specifications that were considered when developing fixed cutoffs for different fit indices, simulation-based methods were proposed to generate individual cutoffs for specific models and data in real-life applications (Millsap, 2007, 2012; Pornprasertmanit et al., 2013). One implementation of this idea was suggested by McNeish and Wolf (2021). The so-called *Dynamic Fit Index Cutoff* aims to generalize the methods applied by Hu and Bentler (1999). Their basic algorithm, also implemented in a Shiny web-application, first needs the user to specify the hypothesized CFA model with standardized factor loadings as well as the sample size. This information is then used to create an alternative model by adding an extra path with a non-zero coefficient. This perturbed model is then used as a population model in the data generating process to simulate R data sets. The hypothesized model (i.e., the original model without the extra path) is fitted to these simulated data sets, which yields R values for each fit index. Based on these values, an empirical distribution can be specified. This process is repeated using the hypothesized model for data generation. This way, a second set of fit indices and resulting empirical distributions are obtained. The distribution based on the perturbed model represents the behavior of a fit index under misspecification, since the “wrong” model (i.e., the model that should actually be evaluated) is fitted to data that stem from a data generating process with an extra path. The second distribution reflects a situation where the model is correctly specified, that is, the model that is used for data generation, is fitted to the simulated data.^3^ From these distributions, cutoff values (if existing) can be derived that have both acceptable false positive and false negative rates. To control the false negative rate, a percentile of the distribution coming from the data generation using the perturbed models is used (e.g., the 5% -percentile for the RMSEA and the SRMR or the 95% -percentile for the CFI). For simulated data, it is known whether a model is misspecified. Accordingly, a cutoff for each fit index can be chosen so that $\mathrm{Pr}(\mathrm{RMSEA}\leq \mathrm{cutoff}|\mathrm{mode}l_{\mathrm{perturbed}})=0.05$ . The false-positive rate is controlled by applying the same principle to the second distribution based on data simulated using the hypothesized model. For example, using the 5% -quantile of this distribution (i.e., accepting a correctly specified model 95% of the time), $\mathrm{Pr}(\mathrm{RMSEA}\geq \mathrm{cutoff}|\mathrm{mode}l_{\mathrm{hypothesized}})=0.05$ is used to determine the cutoff. Note that the two cutoff values derived this way are usually not identical. Thus, if the first value, for example, for the RMSEA, is lower than the second, it is not possible to distinguish between the “true” and the misspecified model at the prespecified rates. It can be derived from simple probability calculations that the actual error rates are higher than those aimed at when deriving the cutoffs. This also makes sense intuitively—when the RMSEA-cutoff under misspecification is lower than under the true model there is an area of ambiguity, where we would both accept the misspecified and the correctly specified model. As these cutoff values are derived using the model fitted by the researcher, data and model characteristics such as the sample size and factor loadings are automatically correctly specified. One can also see from this example that for specific modeling situations, there might not even be a solution which achieves reasonable error rates. As McNeish and Wolf (2021) demonstrated, this is especially the case for scenarios where the scale reliability and sample size are small.

A similar approach called *ezCutoffs* (Schmalbach et al., 2019) was developed to derive tailored cutoff values for fit indices following the procedure of Hu and Bentler (1999). Contrary to the *Dynamic Fit Index Cutoffs* of McNeish and Wolf (2021), data are simulated for a specified model which is subsequently fitted to this data. Accordingly, *ezCutoffs* focuses on the distribution of the fit indices of correctly specified models and derives the cutoff values from a specific quantile of this distribution. This approach can be compared with null hypothesis significance testing where the test decision is solely based on the distribution of the test statistic under the null hypothesis. Thus, no area of ambiguity where both misspecified and correctly specified models are deemed appropriate exists for this approach. However, the *ezCutoffs* approach does not allow researchers to control the type-II error (as the *Dynamic Fit Index Cutoffs* approach does).

Groskurth et al. (2022) developed a different method to derive individual cutoffs tailored to the application context and empirical data at hand. Other than the two purely simulation-based approaches described earlier, Groskurth et al. (2022), in a first step, repeatedly simulate data using a population model that either correspond to the actual model that should be tested (i.e., treating the hypothesized model as a correctly specified model) or to a slightly altered model that serves as a misspecified analysis model.^4^ In a second step, the receiver–operating characteristic (ROC) curves for a set of fit indices are estimated and well-performing fit indices (i.e., indices that reach a certain performance, e.g., an area under the curve ≥.80 ) are selected. For these fit indices, tailored cutoffs are chosen by optimizing both sensitivity and specificity (or minimizing type I and II error).

## Method

For our review, we scanned the full texts of each publication in *Psychological Assessment* (PA) from 2015 to 2020 for the term “CFA OR confirmatory factor analysis” via *PsycArticles*. It was decided for PA due to its special focus on assessment- and scale validation as well as its broad range of studies using CFAs. Our strategy resulted in 456 initial studies, of which $N_{\mathrm{Studies}}=221$ ended up in the final data set. Included were only studies in which the CFAs were reported in the results section (not appendices, footnotes, or preliminary analyses section), single- or multifactor CFAs were used (i.e., complex SEM, bifactor models, and multigroup specifications were excluded), the number of manifest variables per latent factor could be derived and at least one common fit measure (e.g., CFI or RMSEA) was reported. These criteria were applied because we wanted to keep the models comparable while extracting as much information from the studies as possible.

To gain quantitative insight into how psychologists conduct CFA, we extracted and calculated information on model-characteristics, estimation results and compatibility with recommendations for fit evaluation (e.g., Hu & Bentler, 1999). The specific variables collected were: the number of manifest and latent variables, the number of variables per factor, whether correlations between latent variables and/or correlations among residuals were allowed, whether cross-loadings were specified or an independent clusters model was assumed, the median of primary factor loadings, if the same sample was used for preceding exploratory analyses, the sample size, the estimation algorithm, four common fit measures (CFI, RMSEA, SRMR, and TLI), the *p* value of $\chi^{2}$ -tests, and which justification for cutoff values was cited in the article. Based on this information, we analyzed the compatibility of the model fit with recommendations by Hu and Bentler (1999), Browne and Cudeck (1992), and Schermelleh-Engel et al. (2003). To compute the dynamic cutoffs using the R Shiny application *Dynamic Model Fit* (Wolf & McNeish, 2020), we selected 34 studies that reported standardized factor loadings and used maximum-likelihood estimation (or a modified version of it), as these are prerequisites to obtain unbiased estimates from the simulation. If multiple models or samples were present in the study, we analyzed only the best-fitting model on the largest sample. We focused on “level-1”-misspecifications^5^ (McNeish & Wolf, 2021) which are conditions comparable to those evaluated in Hu and Bentler (1999). For comparison, we also calculated the *ezCutoffs* (Schmalbach et al., 2019) for the same models.

We used *R* (Version 4.2.2; R Core Team, 2021) and the R-packages *apaTables* (Version 2.0.8; Stanley, 2021), *dplyr* (Version 1.0.10; Wickham et al., 2021), *ggplot2* (Version 3.4.0; Wickham, 2016), *papaja* (Version 0.1.1; Aust & Barth, 2020), *shiny* (Chang et al., 2021), and *tinylabels* (Version 0.2.3; Barth, 2022) for all our analyses and to write the article.

## Results

First, we looked at the frequencies of sample size, number of variables per factor, and most common fit indices (i.e., RMSEA, SRMR, CFI, GFI, and TLI) by range for every single model ( N=1011 models from the $N_{\mathrm{Studies}}=221$ ). We oriented the ranges to common cutoffs (e.g., Hu & Bentler, 1999; Schermelleh-Engel et al., 2003) but added more gradations to gain a more detailed insight (e.g., 0,0.03,0.05,0.09,0.11, and >0.11 for SRMR). We also calculated the frequencies of (a) whether independent clusters were assumed or cross-loadings were allowed, (b) whether a different sample was used for preceding models, the same sample was used, or the sample was split, and (c) which estimation method was used. Because there is no clear nomenclature of estimation methods, there often were many different names for the same or highly similar methods. Borrowing from Jöreskog et al. (2016), we allocated the estimation methods to one of the following: weighted least squares (WLS), maximum likelihood (ML), unweighted LS (ULS), generalized LS (GLS), diagonally WLS (DWLS). The results of these analyses are shown in Tables 1 to 10 in the column “All models.” Most notably, for more than 50% ( n=639 ) of the models, a sample size of more than 400 was used; for approximately 25% ( n=252 ) of models the sample size even exceeded 1,000 observations. The two most common fit indices were RMSEA ( n=975 ) and CFI ( n=998 ), the least common was GFI ( n=30 ). It also worth mentioning that for 91.3% ( n=923 ) of all models, an independent clusters assumption was made. When models were fitted subsequently, a new sample to fit each model was used for 70.2% ( n=710 ) of models. Unfortunately, for 24.3% ( n=246 ) of models it was not stated whether a new sample was used, the same sample was used, or the sample was split prior to analysis. Regarding the estimation process, ML (42.9%, n=434 , of which 99 used the *Satorra-Bentler Correction* to account for non-normality) and WLS (43.1%, n=436 ) were the two most common choices as estimation algorithms. It should be mentioned, however, that every method can be shown to be a special case of WLS anyways (Jöreskog et al., 2016).

**Table 1.** Frequencies of Sample Size Ranges for All Models and for the Best Models Per Paper.

Sample size	All models		Best models	
	*N*	%	*N*	%
<100	7	0.70	4	1.80
100-200	105	10.40	27	12.30
201-300	146	14.40	27	12.30
301-400	114	11.30	25	11.40
401-1,000	387	38.30	83	37.70
>1,000	252	24.90	54	24.50

**Table 2.** Frequencies of Item to Factor Ratios for All Models and for the Best Models Per Paper.

Item to factor ratio	All models		Best models	
	*N*	%	*N*	%
[2:1, 5:1]	345	34.10	87	39.50
]5:1, 10:1]	365	36.10	85	38.60
]10:1, 15:1]	166	16.40	28	12.70
]15:1, 20:1]	57	5.60	14	6.40
>20:1	78	7.70	6	2.70

**Table 3.** Frequencies of RMSEA Ranges for All Models and for the Best Models Per Paper.

RMSEA	All models		Best models	
	*N*	%	*N*	%
[0, 0.03]	39	3.90	17	7.70
]0.03, 0.05]	160	15.80	53	24.10
]0.05, 0.08]	409	40.50	102	46.40
]0.08, 0.1]	159	15.70	19	8.60
>0.1	208	20.60	20	9.10
NA	36	3.60	9	4.10

*Note.* RMSEA = root mean square error of approximation; NA = not available.

**Table 4.** Frequencies of SRMR Ranges for All Models and for the Best Models Per Paper.

SRMR	All models		Best models	
	*N*	%	*N*	%
[0, 0.03]	35	3.50	13	5.90
]0.03, 0.05]	158	15.60	40	18.20
]0.05, 0.09]	168	16.60	35	15.90
]0.09, 0.11]	18	1.80	1	0.50
>0.11	41	4.10	2	0.90
NA	591	58.50	129	58.60

*Note.* SRMR = standardized root mean square residual; NA = not available.

**Table 5.** Frequencies of CFI Ranges for All Models and for the Best Models Per Paper.

CFI	All models		Best models	
	*N*	%	*N*	%
[0, 0.80]	127	12.60	6	2.70
]0.80, 0.90]	192	19.00	28	12.70
]0.90, 0.95]	291	28.80	60	27.30
]0.95, 0.97]	172	17.00	43	19.50
>0.97	216	21.40	78	35.50
NA	13	1.30	5	2.30

*Note.* CFI = comparative fit index; NA = not available.

**Table 6.** Frequencies of TLI Ranges for All Models and for the Best Models Per Paper.

TLI	All models		Best models	
	*N*	%	*N*	%
[0, 0.80]	93	9.20	5	2.30
]0.80, 0.90]	135	13.40	18	8.20
]0.90, 0.95]	199	19.70	43	19.50
>0.95	180	17.80	64	29.10
NA	404	40.00	90	40.90

*Note.* TLI = Tucker–Lewis index; NA = not available.

**Table 7.** Frequencies of Assumed Structures for All Models and for the Best Models Per Paper.

Structure	All models		Best models	
	*N*	%	*N*	%
Cl	43	4.30	15	6.80
Simple	923	91.30	196	89.10
NA	45	4.50	9	4.10

*Note.* Cl = cross-loadings allowed; Simple = independent clusters models; NA = not available.

**Table 8.** Frequencies of Sample Strategies for All Models and for the Best Models Per Paper.

Sample	All models		Best models	
	*N*	%	*N*	%
New	710	70.20	139	63.20
Same	29	2.90	10	4.50
Split	26	2.60	13	5.90
NA	246	24.30	58	26.40

*Note.* New = new sample used for subsequent models; Same = same sample used for subsequent models; Split = data set was split; NA = not available.

**Table 9.** Frequencies of Estimation Methods for All Models and for the Best Models Per Paper.

Estimation method	All models		Best models	
	*N*	%	*N*	%
DWLS	27	2.70	3	1.40
GLS	1	0.10	1	0.50
ML	434	42.90	93	42.30
ULS	5	0.50	3	1.40
WLS	436	43.10	82	37.30
NA	108	10.70	38	17.30

*Note.* 99 models were estimated using ML with the Satorra-Bentler correction which made it the most frequently applied correction method for non-normality in this study. DWLS = diagonally weighted least squares; GLS = generalized least squares; ML = maximum likelihood; ULS = unweighted least squares; WLS = weighted least squares; NA = not available.

**Table 10.** Frequencies of Fit Evaluation Methods for All Models and for the Best Models Per Paper.

Fit evaluation	All models		Best models	
	*N*	%	*N*	%
CFI	998	98.71	215	97.73
RMSEA	977	96.64	213	96.82
SRMR	420	41.54	91	41.36
TLI	607	60.04	130	59.09
GFI	30	2.97	11	5.00
Chi2 test	348	34.42	78	35.45
AIC	105	10.39	12	5.45

*Note.* CFI = comparative fit index; RMSEA = root mean square error of approximation; SRMR = standardized root mean square residual; TLI = Tucker–Lewis index; GFI = goodness-of-fit index; AIC = Akaike information criterion.

In a second step, we extracted the “best fitting” model per paper based on CFI and calculated the same frequencies as above for the resulting N=220 models. We decided to filter by CFI because it was the most used fit index. The results are shown in Tables 1 to 10 in the column “Best models.” When comparing the frequencies for only the best models with the frequencies for all models, relative frequencies did not differ noticeably.

### Rules of Thumb

Applying common combinatory rules of thumb for fixed fit index cutoffs, a majority of models shows quite poor model fit. Using a “best model” per study ( N=220 ), 64.50% of the respective models show an “unacceptable” model fit according to the cutoffs derived from Hu and Bentler (1999). Browne and Cudeck (1992) advocate for a less strict evaluation of model fit. Applying their respective cutoffs, 47.60% of the models are deemed to have a “reasonable” model fit, 7.90% are considered “employable,” 35.40% “close,” and only 9% of the models are labeled “unemployable.”Schermelleh-Engel et al. (2003) also make suggestions on default cutoffs for common fit indices but do not combine them to a rule of thumb to classify a model fit as “acceptable,”“reasonable,” or something similar. When using their cutoffs, no fit index indicated “good fit” for 40% of the models, while one fit index showed “good fit” for 21.80% of the models (two fit indices: 21.80%, three fit indices: 13.60%, four fit indices: 2.70%).^6^ Schermelleh-Engel et al. (2003) also provide guidelines for an “acceptable” model fit which is obviously less strict, so a higher percentage of models are deemed to have an appropriate fit under these cutoffs (zero acceptable fit indices: 8.20%, one acceptable fit index: 19.10%, two acceptable fit indices: 30.90%, three acceptable fit indices: 34.10%, four acceptable fit indices: 7.70%).

### Tailored Cutoffs for Fit Indices

Calculating the tailored cutoffs for the 34 selected models for which enough information was presented, the *ezCutoffs* approach yielded cutoffs of $\mathrm{CF}I_{\mathrm{cutoff}}=0.975,\mathrm{RMSE}A_{\mathrm{cutoff}}=0.024$ , and $\mathrm{SRM}R_{\mathrm{cutoff}}=0.050$ on average, while the *Dynamic Model Fit* approach provided less strict cutoffs on average ( $\mathrm{CF}I_{\mathrm{cutoff}}=0.973,\mathrm{RMSE}A_{\mathrm{cutoff}}=0.053$ , $\mathrm{SRM}R_{\mathrm{cutoff}}=0.050$ )^7^ balancing the Type I and Type-II errors. The largest mean absolute difference between the tailored cutoffs of the two approaches is found for the RMSEA ( $\mathrm{MA}D_{\mathrm{RMSEA}}=0.029$ ; compared with $\mathrm{MA}D_{\mathrm{SRMR}}=0.019$ and $\mathrm{MA}D_{\mathrm{CFI}}=0.016$ ). Comparing the empirical fit indices with the respective cutoffs, 18.2% of the evaluated models show a good model fit according to the $\mathrm{CF}I_{\mathrm{ezCutoffs}}$ , 47.6% according to the $\mathrm{SRM}R_{\mathrm{ezCutoffs}}$ , and no model according to the $\mathrm{RMSE}A_{\mathrm{ezCutoffs}}$ . In case of the *Dynamic Model Fit* cutoffs, 14.3% (CFI), 33.3% (SRMR), and 14.3% (RMSEA) of the models are considered to fit the data well.^8^

## Discussion

One reason for researchers to use fit indices instead of the exact model test to evaluate the model fit is the fact that the $\chi^{2}$ -test detects smaller and smaller differences between the empirical and the model-implied covariance matrix with increasing sample size (Steiger, 2007). While in general the higher statistical power that is associated with a greater sample size is desirable, evaluating model fit using a strict null hypothesis “ Σ=Σ(Θ) ” which is unrealistic in real-world settings (Bentler, 2007) will inevitably lead to a rejection of the candidate model when the sample size is large. Individual cutoffs tailored to the specific application context are also impacted by the sample size as they are usually designed to control the type I error rates (e.g, Pornprasertmanit et al., 2021; Schmalbach et al., 2019) and become smaller in case of “badness-of-fit” measures (e.g., the RMSEA) or larger in case of goodness-of-fit measures (e.g., CFI) with increasing sample size. Hence, while tailor-made cutoffs promise to prevent misinterpretations and overly optimistic evaluations compared to fixed cutoffs that were derived for rather specific data conditions and model specifications, larger samples foster more extreme cutoffs and more likely result in model rejection, even if the actual misfit is negligible. In small samples though, the tailored *ezCutoffs* (Schmalbach et al., 2019) seem to be too moderate as they tend to support candidate models whose fit would have been deemed not appropriate by common cutoffs (e.g., Hu & Bentler, 1999) or tailored cutoffs that take into account type II error rates (e.g, Groskurth et al., 2022; McNeish & Wolf, 2021). This shows that the *ezCutoffs* approach lacks power in small sample scenarios.

As individual cutoffs also depend on the sample size and seem to perform reasonably well only with moderate sample sizes (i.e., n∈[300;700] ), researchers might have to dismiss the idea of exact model fit (for detailed discussion, see, for example Bentler, 2007; MacCallum, 2003) and rely on a close-fit assessment instead. Moshagen and Erdfelder (2016) discussed this idea arguing that an assessment of close-fit instead of exact fit, which better reflects real-world phenomena anyway, might be preferable as it does not penalize larger samples, but rather makes use of an increase in statistical power. This idea of close fit has been discussed by several authors before (e.g., Browne & Cudeck, 1992; MacCallum, 2003) and was also part of the development of the RMSEA (Steiger, 1998). Moshagen and Erdfelder (2016) build their model evaluation strategy on this premise and the Neyman-Pearson idea of hypothesis testing fulfilling the applied researchers’ wish for a dichotomous decision whether a model fits the data or not. Following Moshagen and Erdfelder (2016), researchers have to choose a minimal effect (i.e., the minimal amount of misfit) they try to detect as all smaller deviations from the data are deemed practically irrelevant. This way, a higher sample size is beneficial as the statistical power to detect meaningful deviations increases, but no discrepancies below that threshold result in rejecting the model. By evaluating the performance of the model fit indices and taking into account Type II error rates, Groskurth et al. (2022) promise to assess the model fit rather independently from the actual sample size similarly to the approach of Moshagen and Erdfelder (2016). The latter may appeal to researchers as they probably prefer choosing a critical value for close-fit (e.g., RMSEA=.03 ), rather than using the ROC curve to derive a cutoff that balances Type I and Type II error rates for a given setting where an alternative model specification has to be provided as well. The close-fit approach of Moshagen and Erdfelder (2016), however, relies on the known $\chi^{2}$ -distribution which makes it easy to calculate but limits its applicability to related indices such as the RMSEA or the GFI as Groskurth et al. (2022) pointed out.

An alternative to the close-fit approach of Moshagen and Erdfelder (2016) and the simulation-based methods by McNeish and Wolf (2021) or Groskurth et al. (2022) is equivalence testing as proposed by Yuan et al. (2016). The basic idea is to determine a so-called *T-size* which describes the minimum tolerable amount of model misspecification for a specific context (e.g., an empirical study). This *T-size* can be related to every common fit index that a researcher usually uses to quantify model (mis-)fit (Yuan et al., 2016). Accordingly, instead of testing the rather unrealistic null hypothesis that the proposed model exactly represents the population model (i.e., the $\chi^{2}$ -test assuming the model-implied covariance matrix to exactly reproduce the population covariance matrix, see Bentler, 2007), equivalence testing evaluates whether the level of misspecification is smaller than a predefined value that represents tolerable or even negligible deviations from the true model. In doing so, the method also allows researchers to determine cutoffs for all common fit indices that take into account the sample size n and the model complexity in terms of the model’s degrees of freedom df . Yuan et al. (2016), for example, present adjusted cutoffs to distinguish “excellent, close, fair, mediocre and poor” model fit given n and df that are adapted from a point-estimate-based rule by Steiger and Lind (1980). K. M. Marcoulides and Yuan (2017) provide a detailed walk-through example on how to use RMSEA- and CFI-based equivalence testing to evaluate model fit beyond the conventional comparisons of descriptive fit indices and arbitrary cutoffs. In comparison to the *Dynamic Model Fit* approach, adjusting cutoffs with the equivalence testing method is less computationally expensive and therefore faster (no data have to be simulated). However, the approach only takes the sample size and the degrees of freedom of the proposed model into account when calculating the adjusted cutoffs, while the simulation-based methods (e.g., *Dynamic Model Fit*) also consider the model dimensionality, the amount of overdetermination, and the communalities of the items (e.g., McNeish and Wolf, 2021). The *T-size*, on the contrary, is calculated for each model individually and takes the specific model characteristics into account (Yuan et al., 2016).

When relying on individual cutoffs, it becomes apparent that often CFI and SRMR show acceptable model fit while the RMSEA is higher than the tailored cutoff indicating poor model fit. Hu and Bentler (1999) showed that the different fit indices are sensitive to different types of misspecifications or model misfit. The SRMR signals misfit regarding the between-factor correlations, whereas the RMSEA focuses more strongly on misspecified loading patterns. Given the high percentage of articles that focus on models with a strict form of simple structure assumption (i.e., independent cluster models), it does not come as a surprise that it is often the RMSEA that questions the model fit for most of the evaluated models in our study. The common assumption that each indicator can be assigned one latent factor and substantial cross-loadings do not exist is quite appealing to researchers as it facilitates the interpretability of the factor model. However, this focus on overly simplified models is typically one reason why measurement models that were developed using exploratory factor analysis cannot be successfully replicated in subsequent CFAs (e.g., Hopwood & Donnellan, 2010; Sellbom & Tellegen, 2019). Hence, researchers should probably consider “imperfect” measurement models with substantial cross-loadings, this might hamper the interpretability of their models because a model with poor fit to the data potentially yields severe misinterpretations.

In this study, we can observe a tendency of models with higher overdetermination (i.e., more indicators per latent factor) to fit the data worse compared to models with a smaller item-to-factor ratio. This pattern of fit indices indicating worse fit for larger and more complex models was also found in simulation studies (e.g., Shi et al., 2019) and can be seen as a weak point of the discussed fit measures. The worse fit in this study could be caused by actual misfit, though, as it appears quite obvious that oftentimes high overdetermination may come at a price of some less suitable indicators. However, this should not be seen as a call for using less indicators per factor to reduce model complexity and artificially increase model fit. Higher overdetermination is in fact associated with higher estimation stability and fewer convergence problems (e.g., Gagne & Hancock, 2006) and usually with higher reliability estimates (e.g., Fabrigar et al., 1999). Besides psychometric considerations regarding the model fit, researchers have to ensure content validity and should therefore be very careful when reducing the number of indicators per factor (e.g., Goretzko et al., 2021).

When focusing on the best-fitting model of each article and comparing the reported fit indices to common cutoff recommendations (Browne & Cudeck, 1992; Hu & Bentler, 1999; Schermelleh-Engel et al., 2003), it has to be stated that several measurement models cannot be considered suitable for the empirical data. Given the usually more strict tailored cutoffs (e.g., the *Dynamic Model Fit* cutoffs) that should be preferred over fixed cutoffs, the comparable poor model fit of some measurement models has to be critically analyzed. As discussed earlier and debated by several authors (e.g., Hopwood & Donnellan, 2010; Marsh et al., 2009), interpretable, yet overly simplistic independent clusters models (i.e., models with an item complexity of one which means that each indicator is only allowed to load on a single factor) are probably responsible for the largest part of the model misfit. Another problem with how researchers usually develop measurement models may be found in earlier stages of the construction process. Goretzko et al. (2021) report that a majority of EFAs still rely on outdated factor retention criteria such as the infamously subjective Scree test or the eigenvalue-greater-one-rule to determine the number of latent factors, even though simulation studies have repeatedly shown that these methods do not provide accurate estimates for the dimensionality of a latent concept (e.g., Auerswald & Moshagen, 2019; Goretzko & Bühner, 2020, 2022; Zwick & Velicer, 1986). If the dimensionality assessment in a preceding EFA has been flawed, it is no surprise that a confirmatory model building on the respective EFA results does not fit empirical data well (e.g., Fabrigar et al., 1999). In combination with traditionally low item reliabilites and high measurement errors in psychological questionnaire data (Gnambs, 2015) and questionable measurement practices (Flake & Fried, 2020), poorly fitting measurement models may severely distort empirical findings and hamper the progress of certain research areas. As the vast majority of models were fitted to categorical data (usually Likert-type items of questionnaires), the actual model fit might be even worse than indicated by common fit indices (Savalei, 2021). In addition, researchers should also keep in mind that cutoffs stemming from simulation studies such as the ones proposed by Hu and Bentler (1999) or tailored cutoffs that are created by the approach of McNeish and Wolf (2021) usually only consider normally distributed data and ML estimation. Hence, the actual model misfit could be even higher for many of the models in our study.

To improve the measurement models that lack model fit, more researchers might consider using modification indices (MI, Saris et al., 1987) to revise their models (as authors indicated to use MI only in 19.1% of the studies in our review). Whittaker (2012) thoroughly describes how MI and related measures such as the expected parameter change (Saris et al., 1987) can be used iteratively to revise the model specification and improve model fit. This so-called specification search can be quite tedious so automated approaches using optimization algorithms have been discussed (G. A. Marcoulides & Drezner, 2003; G. A. Marcoulides et al., 1998). More recently, an automated specification search based on a combination of Tabu search (see also G. A. Marcoulides et al., 1998) and ant-colony optimization (see G. A. Marcoulides & Drezner, 2003) was developed (Jing et al., 2022). When engaging in specification search (especially when automating the procedure), and therefore shifting from confirmatory to exploratory analyses, researchers have to be aware of the risk of over fitting their models to the data (MacCallum et al., 1992). Accordingly, models that were derived from specification search procedures should always be validated on new data to ensure robustness of the measurement model.

While we want to urge psychologist to take the model evaluation of CFA models seriously by using tailored cutoffs instead of fixed cutoffs that do not reflect the respective data conditions and to revise poorly fitting measurement models by getting rid of independent clusters models or at least by reducing the focus on independent clusters factor patterns, we also want to emphasize that model misfit is normal to some degree and that we will not be able to develop perfectly fitting models. Depending on the application context of a scale or questionnaire, exact model fit might be less important and researchers can choose a rather moderate criterion for close model fit (Moshagen & Erdfelder, 2016). Especially in cases where the accuracy of an individual measurement is of little interest, for example, when group means are compared or predictions of an outside criterion at group-level are evaluated, a slightly misspecified model (that only closely fits the data according to an adjusted cutoff for evaluation) can still be useful to gain some insights. In assessment settings, however, where individuals are diagnosed and categorized based on a psychological measure, higher standards have to be in place. Either way, when encountering model misfit, researchers should always investigate the reasons for discrepancies between model predictions and observed data (Hayduk, 2014).

All in all, this study shows that many articles do not report all the necessary results to conduct a “re-analysis” of the model fit with one of the individual cutoff approaches. Especially the rather small number of studies reporting the full loading matrices has to be criticized. Without detailed information on the loading matrices, the respective measurement models can hardly be interpreted by readers or reanalyzed as in our case. Hence, we want to advocate for more thorough and detailed reporting standards for CFA. A transparent depiction of all model parameters (i.e., factor loadings, between-factor correlations, residual variances, correlations among residuals) as well as a comprehensive model evaluation should always be part of a paper that reports a CFA or an SEM in general. The *Journal of the Society for Social Work and Research*, for example, has developed rather strict rules how EFA and CFA results have to be presented (Cabrera-Nguyen, 2010).

## Notes

1. A saturated model is a model without any degrees of freedom that is able to fully reproduce the empirical variance-covariance matrix and can be seen as the benchmark for the best possible model fit.

2. As GFI and NFI, contrary to the CFI, do not include the degrees of freedom, it is very unlikely to obtain a value of one which can only occur if the $\chi^{2}$ -value is exactly zero. Even if the model fits the data well, the $\chi^{2}$ -value is expected to be around the degrees of freedom and not zero.

3. The hypothesized model is treated as if it were correctly specified, the perturbed model that is used to determine the distribution of the fit index values under misspecification might actually be the model that is closer to the true population model. Using the hypothesized model as the starting point for the data simulation makes the approach comparable to the idea of Hu and Bentler (1999)—more detailed information on how their method compares to the simulations of Hu and Bentler (1999) can be found in the original article by McNeish and Wolf (2021).

4. Again, as for the *Dynamic Fit Index Cutoffs* by McNeish and Wolf (2021), treating the hypothesized model as the correctly specified model and using it as the data-generating model in the simulation does not mean that the altered model that is the basis for the misspecified data conditions cannot be closer to the real population model.

5. The *Dynamic Model Fit* approach considers different levels of misspecification. Depending on the model complexity (i.e., the number of latent factors in the CFA model) the number of misspecified paths varies. McNeish and Wolf (2021) describe how their “level-1”-misspecification is comparable to the type of misspecification considered by Hu and Bentler (1999)—a cross-loading is added to one pair of factors in the alternative “misspecified” model, whereas higher levels of misspecification are achieved by adding cross-loadings to different pairs of factors. A concentration of misspecifications on one pair of factors (the authors refer to it as “hyperlocalized misspecification”) would not necessarily lead to a substantively higher increase in the discrepancy function, that is, to a higher overall model misspecification which is why misspecifications are spread out across different pairs of factors (McNeish and Wolf, 2021). Since smaller models (e.g., two-factor models) do not allow for higher level misspecifications, we decided to focus solely on “level-1”-misspecifications for all model evaluations.

6. Note that not all studies report all fit indices, so for some models only two fit indices could indicate a good model fit.

7. For 13 models, not all cutoffs could be derived as areas of ambiguity existed (see also, McNeish & Wolf, 2021).

8. The percentages are smaller for the *Dynamic Model Fit* approach because, for several models that show a good model fit according to the *ezCutoffs* approach, no unique cutoffs could be determined.
